# Classification and Visualization Based on Derived Image Features: Application to Genetic Syndromes

**DOI:** 10.1371/journal.pone.0109033

**Published:** 2014-11-18

**Authors:** Brunilda Balliu, Rolf P. Würtz, Bernhard Horsthemke, Dagmar Wieczorek, Stefan Böhringer

**Affiliations:** 1 Medical Statistics and Bioinformatics, Leiden University Medical Center, Leiden, The Netherlands; 2 Institut für Neuroinformatik, Ruhr-Universität Bochum, Bochum, Germany; 3 Institut für Humangenetik, Universitätsklinikum Essen, Universität Duisburg-Essen, Essen, Germany; University of Ulm, Germany

## Abstract

Data transformations prior to analysis may be beneficial in classification tasks. In this article we investigate a set of such transformations on 2D graph-data derived from facial images and their effect on classification accuracy in a high-dimensional setting. These transformations are low-variance in the sense that each involves only a fixed small number of input features. We show that classification accuracy can be improved when penalized regression techniques are employed, as compared to a principal component analysis (PCA) pre-processing step. In our data example classification accuracy improves from 47% to 62% when switching from PCA to penalized regression. A second goal is to visualize the resulting classifiers. We develop importance plots highlighting the influence of coordinates in the original 2D space. Features used for classification are mapped to coordinates in the original images and combined into an importance measure for each pixel. These plots assist in assessing plausibility of classifiers, interpretation of classifiers, and determination of the relative importance of different features.

## Introduction

In clinical genetics, syndrome diagnosis presents a classification problem, namely whether and if so which syndrome is to be diagnosed for the presenting patient. We here focus on facial image data in order to facilitate this diagnosis. Facial features play an important role in syndrome diagnosis [1]. We have previously demonstrated that information from 2D [2]–[4] images can help in this classification problem. Similar work in 3D, e.g. [5]–[7], confirms this assessment.

This classification problem tends to be high-dimensional, *i.e.* the number of covariates is bigger than the number of observations. Previously, we employed classical dimension reduction by principal component analysis (PCA) and showed that PCA has a large contribution to classification errors [4]. This can be seen by comparing cross-validation (CV) runs used to estimate error once including a PCA within each fold and once performing PCA prior to CV. It is well-known that feature selection must occur within CV to accurately estimate prediction error [8] and indicates that this step plays a crucial role in our application. Principal components (PCs) can exhibit high variation in small data sets [9] which is a possible explanation for our results. To test this assumption, PCA is compared to low-variance transformation and their classification performance is evaluated.

We here pursue penalized regression techniques that are applicable in the high-dimensional setting and can be applied to data directly without preceding dimension reduction [10]. The process of fitting the regression model itself ensures that the final model is low dimensional and asymptotically only contains true predictors. Furthermore, in the low-dimensional setting, a trade-off between variance of predictors and their unbiasedness leads to improved accuracy (such as measured by classification accuracy or the mean-squared-error) as compared to least-squares regression [11]. One advantage of being able to directly work with high-dimensional data is that the dimensionality of data can be even increased further prior to performing classification. We combine these ideas with geometric properties of our data set by applying low-variance transformations on coordinates that represent features in 2D images. For example, distances are computed between graph vertices depending on only two of them. By contrast, PCs in general depend on all vertices derived from a given 2D image. We evaluate the performance of classifiers resulting from such a strategy.

A second goal is to visualize resulting classifiers. If PCA is used together with a linear classification technique such as linear discriminant analysis (LDA) all transformations leading from one group to another in a two-class classification problem can be represented by a single direction in the original feature space. This can be used to create caricatures by moving data points or means away from each other along this direction [2]. If non-linear transformations are involved visualization becomes more challenging. We develop a general framework that allows to create visualizations that indicate importance of neighborhoods in the original 2D space. We apply this methodology to the original syndrome data.

## Materials and Methods

### Ethics statement

Written informed consent was received from all patients or their wardens and the study was approved by the medical ethical committee of the Universitätsklinikum Essen, Germany. Consent was documented on forms which were reviewed and approved by the medical ethical committee of the Universitätsklinikum Essen, Germany.

### Data

**Figure 1:**
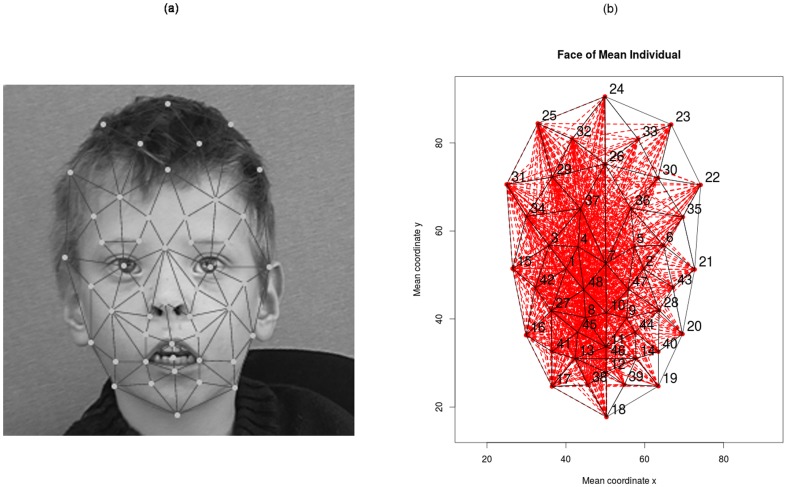
Illustration of data set. (a) Example of registered nodes. (b) Distances between coordinate pairs excluding symmetries. Numbers 1 to 48 correspond to landmarks; red: pairwise edges, excluding symmetries; black: Delaunay triangulation. Example of symmetric distances (25, 24) and (23,24).

Frontal 2D images of 205 individuals each diagnosed with one of 14 syndromes were included in the study. This data set was used in a previous study and is described in detail elsewhere [2]. Table 1 summarizes the number of individuals available per syndrome. In this study, we used coordinates from 48 manually placed landmarks (vertices) that were registered on 2D greyscale images (Figure 1a). These landmarks represent anatomical features in the face. The process of picture pre-processing and landmark registration is described elsewhere [2].

**Table 1:** Description of data set with number of patients per class.

Syndrome	Number of Individuals
Microdeletion 22q11.2 [22q]	25
Wolf–Hirschhorn syndrome [4p]	12
Cri-du-chat syndrome [5p]	16
Cornelia de Lange syndrome [CDL]	17
Fragile X syndrome [fraX]	9
Mucopolysaccharidosis Type II [MPS2]	6
Mucopolysaccharidosis Type III [MPS3]	7
Noonan syndrome [Noon]	13
Progeria [Pro]	5
Prader–Willi syndrome [PWS]	13
Smith–Lemli–Opitz syndrome [SLO]	15
Sotos syndrome [Sot]	15
Treacher Collins syndrome [TCS]	10
Williams–Beuren syndrome [WBS]	42

### Data pre-processing

Vertices were standardized according to translation, rotation and size analogously to a Procrustes analysis [12] (graphs were rotated so that the average angle of symmetric points was 0, the center of the graph was 0 (as defined by the sum of x and y coordinates, respectively) and the size of the graph was scaled to unit size; as defined by the bounding rectangle). On this data, all possible pairwise distances between vertices were computed (D = 1128). To avoid multicollinearity problems, pairs of symmetric distances were averaged (Figure 1b) reducing the number to 778 distances. Using a Delaunay triangulation of the set of averaged vertex positions, we constructed 41 triangles for which 41 areas and 123 angles were computed. Again, symmetric features were averaged. To assess the role of symmetry in syndrome discrimination, asymmetry scores for coordinate pairs, triangle areas and distances were calculated as the sum of squared residuals resulting from the averaging procedure between symmetric information. In order to be able to estimate possible non-linear effects, the square of each feature was also computed. In total, 2×1044 = 2088 covariates were derived per individual from the initial 96 values.

### Statistical Analysis

We performed both simultaneous classification and pairwise classification of syndromes. Simultaneous classification serves to evaluate the problem of assigning a syndrome to a given face, that is, the problem of diagnosis. Pairwise comparisons of syndromes can be used to evaluate similarity of syndromes and to compare the performance achieved with the current data set to other data sets published thus far.

Due to the high dimensionality of the data set (number of individuals  = 205 ≪ number of covariates  = 2088), dimension reduction techniques need to be employed. For simultaneous classification we trained classifiers using regularized multinomial regression with an elastic net penalty [13]. Multinomial regression is a generalization of linear logistic regression model to a multi-logit model, when the categorical response variable has more than 2 levels. For pairwise classification we used regularized logistic regression with an elastic net penalty. Elastic net penalty is a penalized least squares method using a convex combination of the lasso and ridge penalty (with mixing parameter α). In contrast to the LASSO component, which as a general rule selects only one covariate from a group of correlated covariates, the ridge penalty has the effect of distributing effects over covariates that are highly correlated, entering them together into the model. Parameter α can therefore be chosen to control the sparsity of the final model.

We do not consider α to be a tuning parameter but instead consider twenty values of α between 0 and 1 as alternative models. To evaluate model performance, leave-one-out CV was performed. For each of the twenty elastic net models and the PCA analysis, four different covariate sets were used: coordinates of points only, points and their squares, all features and all features and their squared values. Comparisons between these covariate sets allow determining the trade-off between introducing more variation into the data by additional transformations and being able to potentially use more accurate features for the purpose of classification. Fitting an elastic-net model involves choosing a tuning parameter λ for the L_1_-penalty, which was chosen by a nested loop of leave-one-out CV. Likewise, PCA uses an inner CV-loop to estimate principal components (PCs) and train a regression model based on these PCs. In the outer loop, data was mapped to these PCs onto which the prediction model was applied. To directly compare classification performance with a classical PCA approach, the outer CV loop was identical for the elastic net and PCA models, *i.e.* outer CV-folds were computed and identically used for all models.

To compute simultaneous accuracy for the PCA, we trained classifiers using multinomial logistic regression. 70 PCs were extracted from the whole data set. Subsequently, stepwise forward selection was performed to select PCs relevant for the classification decision based on the Akaike information criterion (AIC). The selected models were used to predict the samples in the test set of each CV-fold.

All statistical analyses were performed using the software package R (version 3.0.1 [14]). We used the package *geometry* for the Delaunay triangulation and package *glmnet* to perform model selection and regularized multinomial and logistic regression with an elastic net penalty.

### Visualization

**Figure 2:**
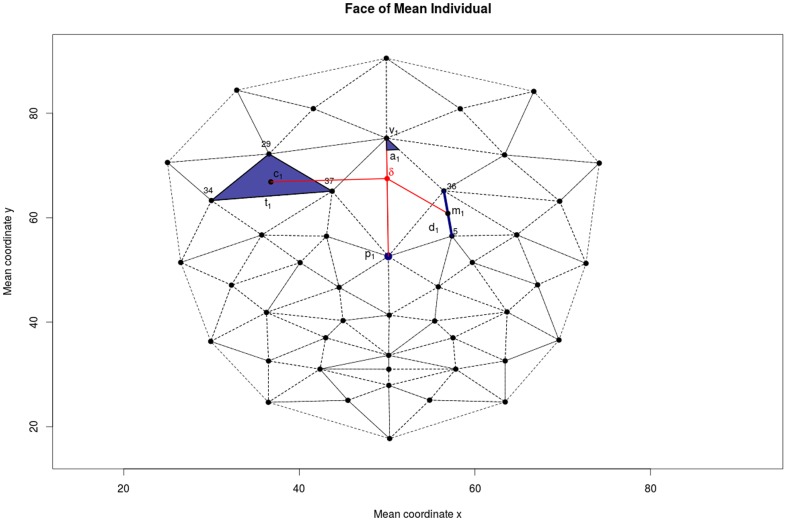
Importance weighting. Illustration of the procedure to compute importance for point δ. Contributions of point p_1_, area of triangle t_1_, distance d_1_, and angle a_1_ (blue) are weighted according to distance to δ (red). Distances to p_1_, centroid c_1_, midpoint m_1_, vertex v_1_ are used for p_1_, t_1_, d_1_, and a_1_, respectively.

The aim of our visualization strategy is to assign an importance value to each point in an average image of a class that represents how important features in that location are to discriminate the given class. While this strategy does not directly represent changes in, for example, distances, it allows to combine all features relevant for a classification decision in a single image. Figure 2 illustrates the process of computing the color coefficient for a point δ based on the following significant features: a point p1, a distance d1, an area of triangle t1 and an angle of a traingle a1. We assume that a weight is assigned to each feature, in our case regression coefficients denoted with β_p1_, β_d1_, β_t1_ and β_a1_. To calculate the importance of point δ we define the distances of this point to the significant features. For p1 we compute the Euclidean distance of δ to p1, for d1 we compute the Euclidean distance of δ to m1, the midpoint of d1, for t1 we compute the Euclidean distance of δ to c1, the centroid of t1 and for a1 we compute the Euclidean distance to c1, the vertex of a1, respectively. The importance of each point is then defined as the sum of the weights, in our case regression coefficients, inversely weighted by the distances. This definition assumes that all weights are measured on the same scale, which can be assured by standardizing covariates in the regression setting. Finally, we normalize these importance values to (0, 1) by using the logistic function and we map resulting values to a color palette. As we symmetrized our data set, we also create symmetrized plots, *i.e.*, one half is computed and mirrored to the other part. We overlay these maps on average facial images for the class corresponding to the respective classifier. The procedure of producing average images is described elsewhere [15].

For *glmnet* we used the regression coefficient of each feature as weights. To obtain the coefficients of each feature when PCA was performed, regression coefficients of PCs are back-calculated to the original feature space using the loadings matrix. The weight for each feature is the sum of contributions over all PCs.

## Results

### Model Selection

**Figure 3:**
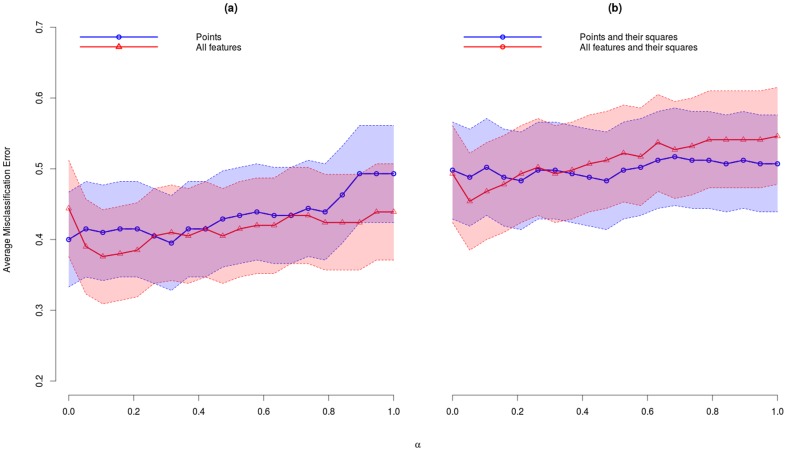
Average misclassification error *glmnet*. Average misclassification error with 95% confidence intervals across leave-one-out cross-validation for models with different values of mixing parameter α. (a) all features (red) and only points (blue) were used and (b) all features and their squares (red) and only points and their squares (blue) were used.

Average misclassification error (AME) rate for each choice of the mixing parameter α and feature set are reported in Table 2. In the last row of the table, we list the results for the PCA. In Figure 3, we illustrate these results together with the 95% confidence intervals. The best model for *glmnet* is obtained for α = 0.11 when the set of all features was used with an AME  = 0.38 (95% CI: 0.31–0.44). PCA performed best when only points were used with AME  = 0.53 (95% CI: 0.46–0.60). The AME of *glmnet* decreased with increasing number of features. In contrast, the AME of PCA increases.

**Table 2:** Average misclassification error (AME) with 95% confidence interval for leave-one-out cross validation for *glmnet*, 20 different values of α (see text), and PCA using only points (p), all features (a), only points and their squares (p+p^2^) and all features and their squares (a+a^2^).

	p	a	p+p^2^	a+a^2^
α = 0	.400 (.333,.467)	.444 (.376,.512)	.498 (.429,.566)	.493 (.424,.561)
α = .05	.415 (.347,.482)	.390 (.323,.457)	.488 (.419,.556)	.454 (.385,.522)
α = .11	.410 (.342,.477)	**.376 (.309,.442)**	.502 (.434,.571)	.468 (.400,.537)
α = .16	.415 (.347,.482)	.380 (.314,.447)	.488 (.419,.556)	.478 (.410,.547)
α = .21	.415 (.347,.482)	.385 (.319,.452)	.483 (.414,.552)	.493 (.424,.561)
α = .26	.405 (.338,.472)	.405 (.338,.472)	.498 (.429,.566)	.502 (.434,.571)
α = .32	.395 (.328,.462)	.410 (.342,.477)	.498 (.429,.566)	.493 (.424,.561)
α = .37	.415 (.347,.482)	.405 (.338,.472)	.493 (.424,.561)	.498 (.429,.566)
α = .42	.415 (.347,.482)	.415 (.347,.482)	.488 (.419,.556)	.507 (.439,.576)
α = .47	.429 (.361,.497)	.405 (.338,.472)	.483 (.414,.552)	.512 (.444,.581)
α = .53	.434 (.366,.502)	.415 (.347,.482)	.498 (.429,.566)	.522 (.453,.590)
α = .58	.439 (.371,.507)	.420 (.352,.487)	.502 (.434,.571)	.517 (.448,.586)
α = .63	.434 (.366,.502)	.420 (.352,.487)	.512 (.444,.581)	.537 (.468,.605)
α = .68	.434 (.366,.502)	.434 (.366,.502)	.517 (.448,.586)	.527 (.458,.595)
α = .74	.444 (.376,.512)	.434 (.366,.502)	.512 (.444,.581)	.532 (.463,.600)
α = .79	.439 (.371,.507)	.424 (.357,.492)	.512 (.444,.581)	.541 (.473,.610)
α = .84	.463 (.395,.532)	.424 (.357,.492)	.507 (.439,.576)	.541 (.473,.610)
α = .9	.493 (.424,.561)	.424 (.357,.492)	.512 (.444,.581)	.541 (.473,.610)
α = .95	.493 (.424,.561)	.439 (.371,.507)	.507 (.439,.576)	.541 (.473,.610)
α = 1	.493 (.424,.561)	.439 (.371,.507)	.507 (.439,.576)	.546 (.478,.615)
PCA	**.532 (.463,.600)**	.810 (.756,.864)	.527 (.458,.595)	.727 (.666,.788)

**Figure 4:**
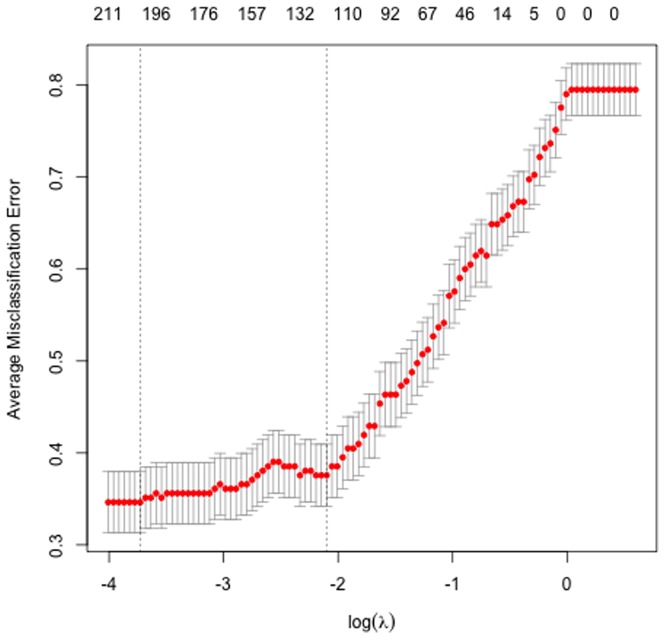
Average misclassification error for values of tuning parameter λ when α = .11.

Results from the inner leave-one-out CV for *glmnet* models for α = 0.11 to choose tuning parameter λ that gives the lowest AME rate are plotted in Figure 4. The lowest AME rate was obtained for λ = 0.047.

The difference between the best glmnet model for all features and best PCA model (points) is significant (Z-test for 2 population proportions, P-value  = .0015).

### Simultaneous classification

Results for simultaneous classification using the best *glmnet* model are reported in Table 3 and 4. Specifically, Table 3 shows breakup of AME per syndrome. The best performance was achieved for WBS (AME  = 9.5%) and 22q (AME  = 20%). The lowest performance was achieved for the syndromes with the smallest sample sizes, MPS2 (AME  = 100%) and MPS3 (AME  = 70%). Table 4 shows the corresponding confusion matrix, *i.e.* what were the classification decisions per syndrome? For example, 22q was confused with 5p, Sot and WBS, whereas MPS2 was confused with MPS3, 22q, SLO and WBS.

**Table 3:** Simultaneous average misclassification error (AME) per syndrome.

Syndrome	AME
22q	0.20
4p	0.58
5p	0.50
CDL	0.53
fraX	0.33
MPS2	1.00
MPS3	0.71
Noon	0.46
Pro	0.40
PWS	0.62
SLO	0.33
Sot	0.33
TCS	0.40
WBS	0.10

**Table 4:** Confusion matrix for the best *glmnet* model, α = .11, using all features.

		Predicted Class													
		22q	4p	5p	CDL	fraX	MPS2	MPS3	Noon	Pro	PWS	SLO	Sot	TCS	WBS
True Class	22q	0.80	0.00	0.12	0.00	0.00	0.00	0.00	0.00	0.00	0.00	0.00	0.04	0.00	0.04
	4p	0.00	0.42	0.00	0.17	0.00	0.00	0.00	0.17	0.00	0.00	0.00	0.17	0.00	0.08
	5p	0.19	0.06	0.50	0.00	0.00	0.00	0.00	0.00	0.00	0.00	0.00	0.06	0.00	0.19
	CDL	0.00	0.00	0.00	0.47	0.18	0.00	0.00	0.06	0.00	0.06	0.06	0.00	0.00	0.18
	fraX	0.00	0.00	0.00	0.11	0.67	0.00	0.00	0.00	0.00	0.00	0.00	0.00	0.00	0.22
	MPS2	0.33	0.00	0.00	0.00	0.00	0.00	0.17	0.00	0.00	0.00	0.33	0.00	0.00	0.17
	MPS3	0.00	0.00	0.14	0.00	0.00	0.00	0.29	0.00	0.00	0.00	0.00	0.14	0.00	0.43
	Noon	0.08	0.08	0.00	0.00	0.00	0.00	0.00	0.54	0.00	0.00	0.00	0.15	0.08	0.08
	Pro	0.20	0.00	0.00	0.00	0.00	0.00	0.00	0.00	0.60	0.00	0.00	0.20	0.00	0.00
	PWS	0.15	0.00	0.08	0.15	0.00	0.00	0.00	0.00	0.00	0.38	0.00	0.00	0.00	0.23
	SLO	0.00	0.07	0.07	0.00	0.00	0.00	0.07	0.00	0.00	0.00	0.67	0.00	0.00	0.13
	Sot	0.13	0.07	0.00	0.00	0.00	0.00	0.00	0.00	0.00	0.07	0.07	0.67	0.00	0.00
	TCS	0.10	0.10	0.00	0.00	0.00	0.00	0.00	0.10	0.00	0.00	0.00	0.00	0.60	0.10
	WBS	0.02	0.00	0.00	0.02	0.00	0.00	0.00	0.00	0.00	0.00	0.05	0.00	0.00	0.90

Rows indicate the percentages of predicted syndromes for each of the syndromes in the study.

We summarize the number of components used for the classification decision in Table 5. Approximately 200 features were selected per syndrome. Distances seemed to be more important (ca. 150 distances per syndrome) as compared to the other features (points between 10 and 25, angles between 20 and 40, < 20 for areas and coordinates).

**Table 5:** Number of non zero coefficients for each syndrome for the best glmnet model (α = .11 using all features).

Syndrome	*t*	*p*	*d*	*ar*	*an*
22q	244	27	157	12	46
4p	204	28	138	9	28
5p	243	26	173	15	28
CDL	200	22	120	13	43
fraX	170	14	106	8	40
MPS2	150	12	99	10	28
MPS3	187	17	118	11	40
Noon	197	17	118	15	46
Pro	150	10	105	6	28
PWS	203	20	144	9	28
SLO	235	20	183	8	21
Sot	220	25	153	9	31
TCS	171	16	111	10	33
WBS	257	19	181	17	38
total	1045	96	778	41	123

*t*: total, *p*: points, *d*: distances, *ar*: areas and an: angles.

### Pairwise classification

Results for pairwise comparisons of syndromic conditions are reported in Table 6, which lists AME. For many pairs, such as FraX/22q or FraX/4p, we achieve an AME of 0%. The highest AME was observed when discriminating between MPS2/MPS3, two syndromes with similar facial appearance (38%).

**Table 6:** Pairwise average misclassification error rate for the best *glmnet* model.

	22q	4p	5p	CDL	fraX	MPS2	MPS3	Noon	Pro	PWS	SLO	Sot	TCS
4p	.05												
5p	.20	.14											
CDL	.05	.00	.09										
fraX	.03	.00	.04	.15									
MPS2	.10	.11	.18	.04	.00								
MPS3	.09	.11	.22	.00	.06	.38							
Noon	.11	.28	.14	.07	.00	.11	.05						
Pro	.03	.12	.05	.00	.00	.00	.00	.00					
PWS	.16	.04	.24	.27	.14	.11	.10	.04	.00				
SLO	.05	.11	.16	.06	.00	.10	.18	.04	.05	.11			
Sot	.02	.19	.19	.00	.00	.10	.05	.14	.00	.04	.07		
TCS	.06	.18	.12	.04	.00	.12	.00	.13	.00	.04	.04	.04	
WBS	.06	.06	.09	.08	.04	.08	.08	.02	.00	.09	.12	.00	.02

### Visualization

**Figure 5:**
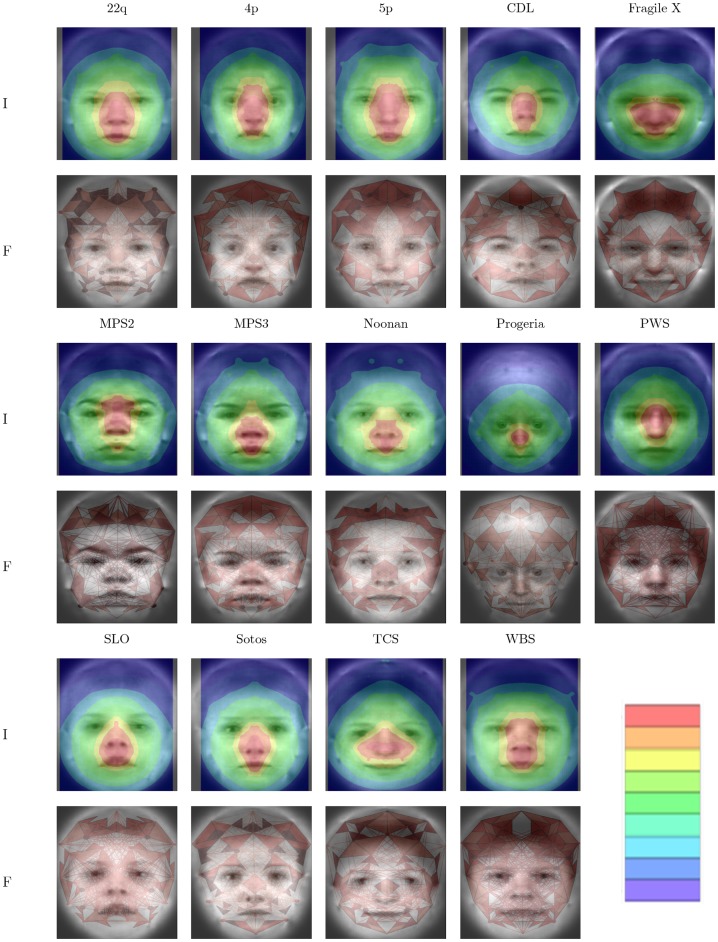
Importance plots *glmnet*. Visualization of simultaneous classification for syndromes. For each syndrome an importance plot (row I) and a plot visualizing classification features (row F) is provided. Importance plot assigns an importance with respect to classification to each point as described in the text. Feature plots visualize absolute regression coefficients by thickness of line segments (distances), size of points (coordinates), color of areas (areas; dark red more important than light red) and small triangles (angles; dark red more important than light red).

**Figure 6:**
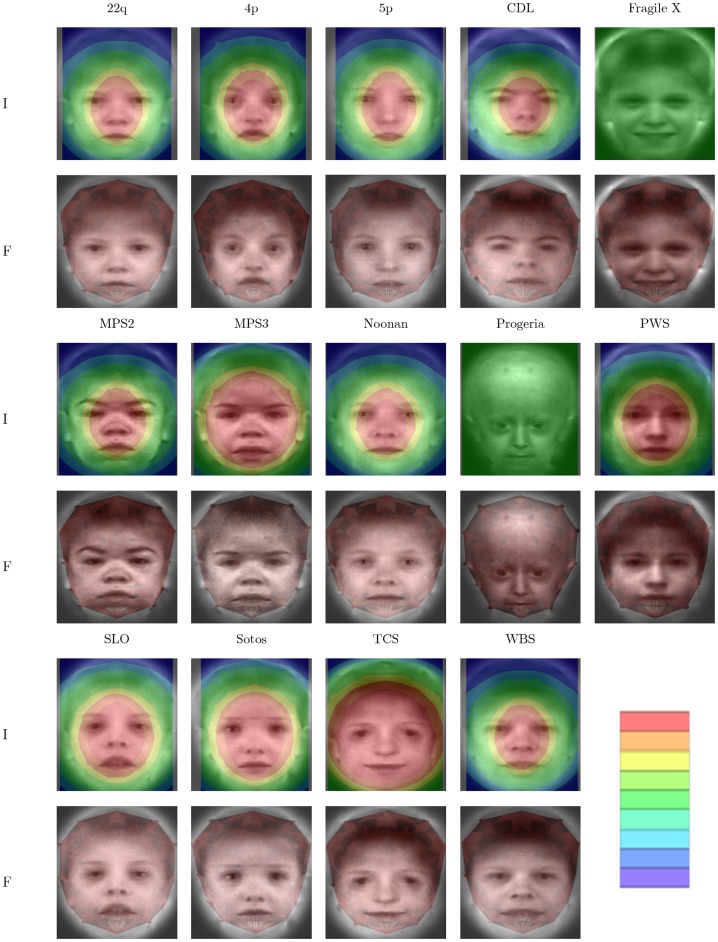
Importance plots PCA. Visualizations analogous to figure 5 for PCA based classification.

Results from the visualization process are depicted in Figure 5 and 6, for best *glmnet* and PCA model, respectively. For these figures, importance below a threshold is ignored to better show the underlying average image. The same color mapping scheme and scale is used for all sub-figures, making colors comparable. As a comparison, features were also visualized by drawing line segments, points, areas, and small triangles to visualize the importance of distances, coordinates, areas, and angles, respectively. In supplementary images we provide importance plots for the different data components.

All visualizations show distinct patterns of important regions in the face. In general, the central part of the face is included for all syndromes. As an example, progeria is described to exhibit midface hypoplasia and micrognathia (MIM #176670 [16]) thus featuring a relatively enlarged forehead. Overall importance is focused around the nose whereas the coordinate component shows importance in forehead regions as well as the nose (Figures S1, S2 and S3), a finding that is discussed below.

## Discussion

Dimension reduction can pose a formidable problem in classification problems if data sets are small. It is well known that methods like PCA can induce big additional variation in data sets thereby reducing classification accuracy. Partly in response to problems like this, penalized regression techniques were developed to estimate classifiers that trade unbiasedness (*i.e.*, parameter estimates that are correct on average) for more stable estimation of classifiers (as measured by the variance of parameter estimates) [10], [11]. We have used these ideas in the current study and demonstrate that additional data transformations can even improve classification accuracy. We chose data transformations with low variance as compared to variation of PCs. If these derived features better describe differences between groups, the tradeoff (more variation, more accurate features) can result in a net benefit in terms of classification accuracy, as was the case in this study. As a conclusion, carefully chosen data transformations that increase dimensionality of data sets can improve classification accuracy even if a problem is already high-dimensional. Which transformations to choose is data set specific. As a general rule, each transformation should only depend on few original features (*e.g.*, distances, angles, areas in our case depend on maximally 6 coordinates) in contrast to many (PCA at the other extreme).

Pair-wise classification results can be used to get exploratory insights. For example, the pair MPS2/MPS3 has an AME close to 40% implying that the features used in this study do not allow to distinguish this pair of syndromes. In the genetic context, pair-wise classification accuracies can be used as a descriptive measure of phenotypic distinctness.

Our attempt at visualization has the advantage of being generic. As long as a distance of a feature with a point can be defined, we can apply this approach and produce images representing importance of image neighborhoods for the classification decision. At the same time this is a disadvantage as no distinction is made between different types of features and it is impossible to derive such information from our images in general. This shortcoming can be partly addressed by visualizing different data components, which might give important additional information. For example, in the progeria example mentioned above, the nose was visualized as the most important feature in this data set. A narrow nose bridge is a distinguishing feature for progeria in our data set, however, visualizing coordinates and angles alone also indicates the forehead as a selected feature for this syndrome which would be a more expected feature from the genetic perspective. It is therefore possible to get a better understanding of classifiers by means of such stratified importance plots.

A related problem is that in high-dimensional problems penalized methods have to be selective and choose few features for the final model from the set of all input features. This can well lead to the omission of features that are more easily recognized by human raters. We tried to mitigate this problem by two approaches. First, by using elastic net regression we tried to create less sparse models, thereby retaining more features as compared to a pure LASSO. As a striking example, had we not symmetrized our data, the LASSO would have ignored one of the highly correlated symmetric features whereas elastic net (for an appropriate value of α) would have split the effect almost equally between the two. Second, our means of creating importance plots takes into account the locality of features. If two distances share one vertex, and their vectors are not linearly independent, they are likely to be correlated. Even if one of the distances would be omitted from the model its importance would still be mapped through the correlated distance that shares close proximity.

It follows that the best performing classifier is not necessarily the most intuitive to visualize and we accept that our approach has limitations in overcoming all possible difficulties. Yet, we believe that the visualizations presented here have several merits. First, plausibility of classifiers can be checked. In our case the more variable positions in the hair should be less likely to be important as is the case. Second, these visualizations could be used to refine data pre-processing. In our case we could decide to omit coordinates from the upper rim of the graph altogether, as they do not appear to be important. Third, these visualizations can make it more easy to interpret the actual regression models and can potentially lead to deeper insights for the data expert, in our case the clinical geneticist.

Finally, it is challenging but possible to produce actual caricatures, which would overemphasize images features relevant for the classification decisions. Such caricatures would have to account for the potentially selective nature of the model selection discussed above and presents a computational problem due to the high dimensionality of the feature space (D = 2088 in our case). We intend to pursue such an approach.

## Conclusions

In conclusion, we have demonstrated the importance of small variance transformations in classification problems of facial data to improve accuracy. Visualization and interpretation remains challenging and can be guided by importance plots that can summarize highly complex classifiers in a single figure or few figures.

## Supporting Information

Figure S1 **Visualization of simultaneous classification for syndromes.** For each syndrome importance plots of different data components are shown. This figure contains syndromes 22q, 4p, 5p, CDL, and Fragile X.
(TIFF)

Figure S2 **Visualization of simultaneous classification for syndromes.** For each syndrome importance plots of different data components are shown. This figure contains syndromes MPS2, MPS3, Noonan, progeria, and PWS.
(TIFF)

Figure S3 **Visualization of simultaneous classification for syndromes.** For each syndrome importance plots of different data components are shown. This figure contains syndromes SLO, Sotos, TCS, and WBS.
(TIFF)
