# Supplementary figures and images for: Classification and Visualization Based on Derived Image Features: Application to Genetic Syndromes
Source: PLoS One. 2014 Nov 18;9(11):e109033. doi: 10.1371/journal.pone.0109033 (PMC4236018; doi:10.1371/journal.pone.0109033)

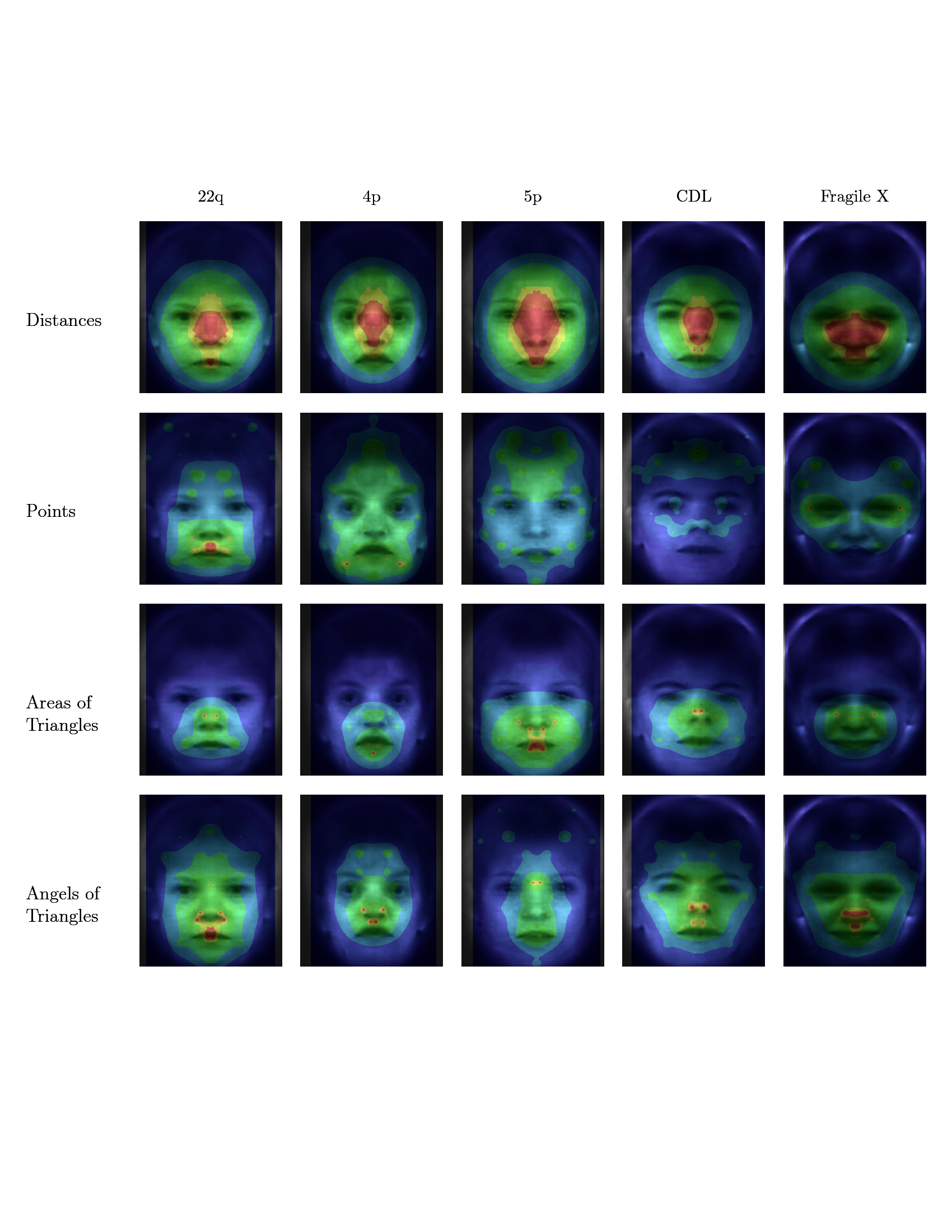

Supplement: Figure S1 — Visualization of simultaneous classification for syndromes. For each syndrome importance plots of different data components are shown. This figure contains syndromes 22q, 4p, 5p, CDL, and Fragile X. (TIFF) [file pone.0109033.s001.tiff]

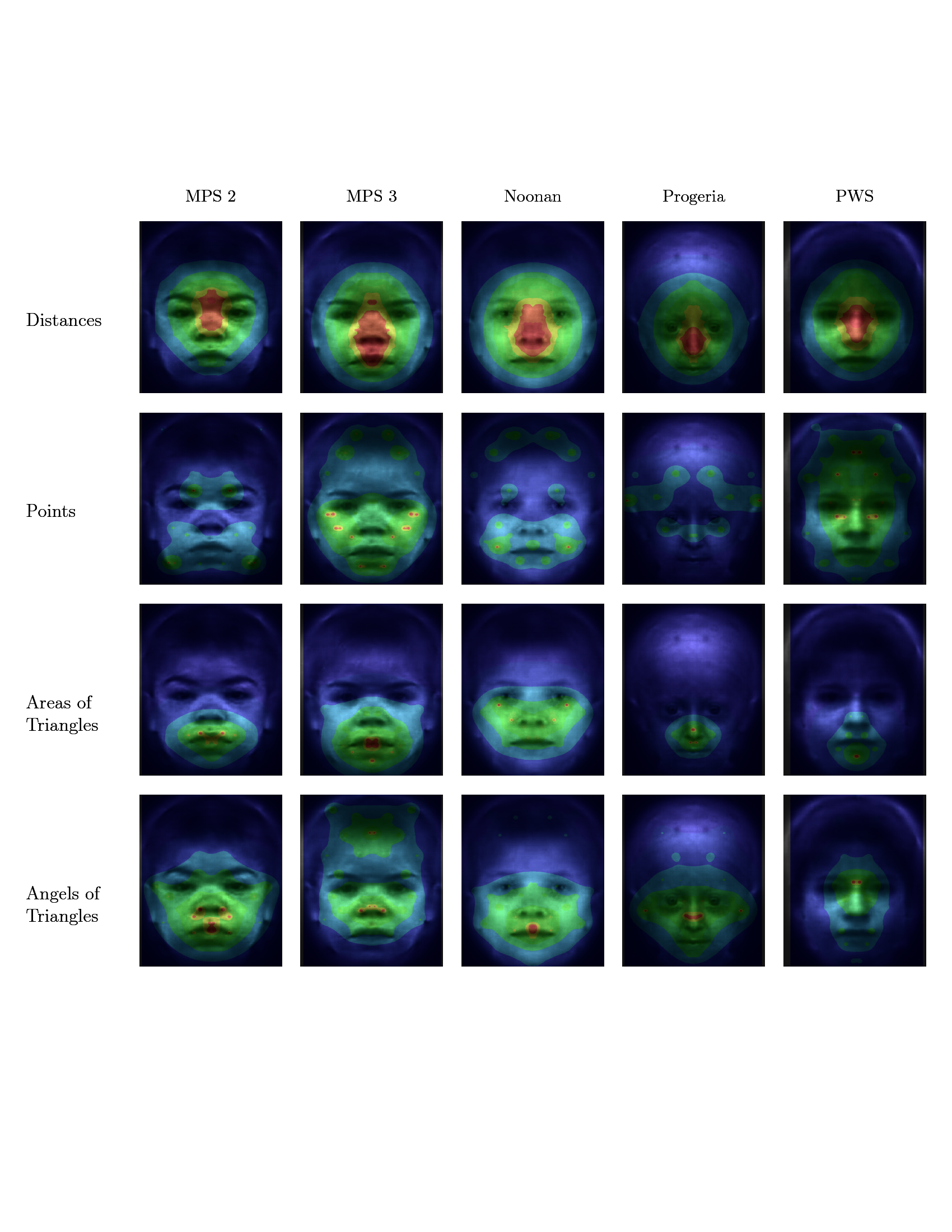

Supplement: Figure S2 — Visualization of simultaneous classification for syndromes. For each syndrome importance plots of different data components are shown. This figure contains syndromes MPS2, MPS3, Noonan, progeria, and PWS. (TIFF) [file pone.0109033.s002.tiff]

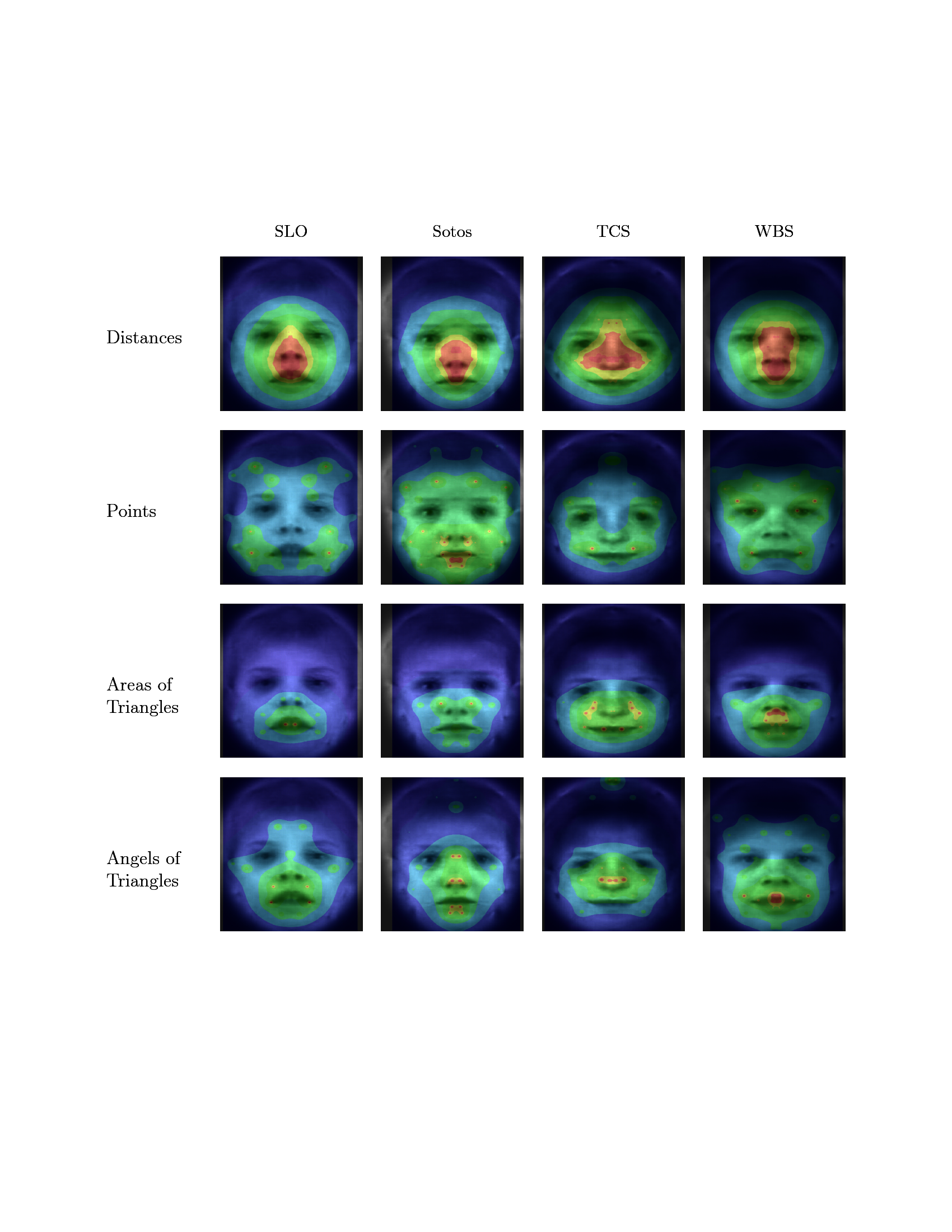

Supplement: Figure S3 — Visualization of simultaneous classification for syndromes. For each syndrome importance plots of different data components are shown. This figure contains syndromes SLO, Sotos, TCS, and WBS. (TIFF) [file pone.0109033.s003.tiff]
